# Brazilian multicenter study on prevalence of preterm birth and associated factors

**DOI:** 10.1186/1471-2393-10-22

**Published:** 2010-05-19

**Authors:** Renato Passini, Ricardo P Tedesco, Sergio T Marba, Jose G Cecatti, Ruth Guinsburg, Francisco E Martinez, Marcelo L Nomura

**Affiliations:** 1 Department of Obstetrics and Gynecology, School of Medical Sciences, University of Campinas, Brazil; 2 Neonatology Unit, Department of Pediatrics, School of Medical Sciences, University of Campinas, Brazil; 3 Department of Pediatrics, Federal University at São Paulo, Brazil; 4 Department of Pediatrics, State University at Ribeirão Preto, Brazil

## Abstract

**Background:**

The occurrence of preterm birth remains a complex public health condition. It is considered the main cause of neonatal morbidity and mortality, resulting in a high likelihood of sequelae in surviving children. With variable incidence in several countries, it has grown markedly in the last decades. In Brazil, however, there are still difficulties to estimate its real occurrence. Therefore, it is essential to establish the prevalence and causes of this condition in order to propose prevention actions. This study intend to collect information from hospitals nationwide on the prevalence of preterm births, their associated socioeconomic and environmental factors, diagnostic and treatment methods resulting from causes such as spontaneous preterm labor, prelabor rupture of membranes, and therapeutic preterm birth, as well as neonatal results.

**Methods/Design:**

This proposal is a multicenter cross-sectional study plus a nested case-control study, to be implemented in 27 reference obstetric centers in several regions of Brazil (North: 1; Northeast: 10; Central-west: 1; Southeast: 13; South: 2). For the cross sectional component, the participating centers should perform, during a period of six months, a prospective surveillance of all patients hospitalized to give birth, in order to identify preterm birth cases and their main causes. In the first three months of the study, an analysis of the factors associated with preterm birth will also be carried out, comparing women who have preterm birth with those who deliver at term. For the prevalence study, 37,000 births will be evaluated (at term and preterm), corresponding to approximately half the deliveries of all participating centers in 12 months. For the case-control study component, the estimated sample size is 1,055 women in each group (cases and controls). The total number of preterm births estimated to be followed in both components of the study is around 3,600. Data will be collected through a questionnaire all patients will answer after delivery. The data will then be encoded in an electronic form and sent online by internet to a central database. The data analysis will be carried out by subgroups according to gestational age at preterm birth, its probable causes, therapeutic management, and neonatal outcomes. Then, the respective rates, ratios and relative risks will be estimated for the possible predictors.

**Discussion:**

These findings will provide information on preterm births in Brazil and their main social and biological risk factors, supporting health policies and the implementation of clinical trials on preterm birth prevention and treatment strategies, a condition with many physical and emotional consequences to children and their families.

## Background

Preterm birth is a major cause of neonatal morbidity and mortality worldwide. Classically defined as the birth that occurs before the 37^th ^week of pregnancy [1], it is also the main responsible for deficiencies acquired after birth. Except for congenital malformations, 75% of perinatal deaths and 50% of neurological abnormalities are directly attributed to preterm [2-4].

Thanks to advances in technology and improvements in health care, several preterm newborn infants survive with least sequelae. However, many of them remain vulnerable to long term complications that may persist all over their lives. Among the main resulting morbidities are neurosensory deficits (blindness, deafness), necrotizing enterocolitis, intraventricular hemorrhage, and delay in physical and mental development [5,6].

In a recent publication, the United States' Institute of Medicine reported that the incidence of preterm birth has increased in the last two decades [7]. Preterm newborn infants represented 9.4% of live births in the United States in 1981. In 2004, this proportion increased to 12.5% [8]. Other data from the United States confirmed these figures, showing that, in 2006, 12.8% of births were preterm, which represented a 21% increase compared to 1990 [4]. This fact has greatly motivated the interest from authorities and those responsible for the different sectors of maternal-infant health, either public or private, in several countries of the world.

Preterm births are spontaneous in 75% of the cases [9]. Of those resulting from medical indication, more than half are associated with pre-eclampsia, fetal distress, intrauterine growth restriction, abruptio placentae, and placental insufficiency [10]. Although the preterm birth etiology is heterogeneous, it has known associated risk factors. Notable among these factors are previous spontaneous preterm labor, low socioeconomic level, and the interaction between genetic and environmental factors.

A relevant aspect is the relationship between preterm birth and the presence of fetal and maternal infections. It is estimated that approximately half the spontaneous preterm births are associated with intrauterine infection, which triggers the maternal and fetal inflammatory reaction, leading to the occurrence of uterine contractions and preterm labor [11]. In addition, the severity of neonatal complications is higher in newborn infants from mothers with intraamniotic infection [12]. Study carried out in the state of São Paulo established an association between positive cervical cultures and maternal and fetal infectious morbidities, such as urinary tract infections and neonatal infections, especially in cases of preterm [13]. There are many studies trying to associate infections such as periodontal disease and bacterial vaginosis with preterm labor and prelabor rupture of membranes. However, results are still inconclusive [14,15].

Prelabor rupture of membranes has a close relationship with preterm birth. It is estimated that it is responsible for up to 30% of all preterm births [16]. Although it frequently occurs at term, when it occurs preterm it mostly results in preterm labor. A hypothesis is that the same infectious mechanisms that cause the loss of membrane integrity are responsible for triggering the inflammatory process that results in uterine contractility. Therefore, according to this interpretation, they would be two clinical manifestations of the same infectious condition [17].

Also, there is strong relationship between preterm birth and multiple pregnancies, and prematurity is the main complication in these pregnancies. Among the reasons for this association is the early and exaggerated stretching of myometrial fibers, although extensive researches try to determine the physiopathological mechanism that explains this event, as well as to establish screening tests and preventive measures in order to avoid them [18].

Therefore, the probable causes of preterm birth can be divided in three major groups: spontaneous, therapeutic interruption of pregnancy, and prelabor rupture of membranes.

The prevalence of preterm birth in Brazil in 2006 was 6.5% [19]. However, this number may not be real. Population-based studies demonstrate that it is higher [20]. The unreal estimate from governmental bodies may be a consequence of difficulties to accurately estimate the gestational age, difficulties in information systems that may result in poor records, therefore decreasing their reliability, and the significant population differences in a continental-sized country. In addition, as in other countries, this prevalence may have increased in the last couple of years, which has not been appropriately emphasized. Late or sometimes nonexistent prenatal care makes it difficult or even impossible to provide a reliable estimate of the gestational age. The same is true regarding the lack of neonatal care during labor, which also contributes to an inaccurate estimate of the gestational age and, consequently, of the incidence of preterm in the country. In addition to easy and appropriate access to prenatal care, it is imperative to develop a national standard to establish the gestational age through the evaluation of the newborn infant, which is essential to implement guidelines in different clinical situations of the obstetrics and pediatrics practices.

In view of all these considerations, we conclude that it is important to assess the situation of preterm birth in Brazil, knowing its real prevalence and associated socioeconomic factors, adopted preventive measures, diagnostic and screening methods applied, interventions, and short term and long term maternal and neonatal results, so that, in association with other developed countries, this evidence will guide health professionals and policy makers in applying the necessary preventive and appropriate measures to face this problem.

The general objective of this study is to evaluate the prevalence of preterm births in several hospitals in Brazil, determining its main causal factors, associated risk factors, treatment protocols, and perinatal morbidity and mortality. Specific objectives are:

1. To know the prevalence of preterm birth in 27 institutions of different Brazilian regions, identifying the methods used to determine the gestational age at birth;

2. To identify and quantify the main causes of preterm birth in these institutions;

3. To identify the diagnostic criteria used by these institutions to identify preterm birth causes;

4. To identify and quantify the main factors associated with preterm birth causes in the different institutions, comparing with term birth;

5. To identify, describe and group the different standards used by these institutions to treat preterm birth causes;

6. To evaluate the preterm birth treatments in these institutions;

7. To determine the early and late neonatal results of preterm births occurred in these institutions.

## Methods

### Study design and location

A multicenter study will be implemented in 27 reference obstetric units from several Brazilian regions (Appendix 1) under the coordination of the Department of Obstetrics and Gynecology of the School of Medical Sciences, University of Campinas, São Paulo, Brazil. For a period of six months, researchers will perform a prospective surveillance and data collection to identify preterm birth cases, their main causes and consequences. A first step consisting of people training and database development will take place prior to the collection of data. During the period of data collection, two kinds of study will be carried out: a cross-sectional study to assess the preterm rate in each participant center, identifying preterm levels like late preterm (34 - 36.6 weeks), early preterm (32 - 33.6 weeks), and extreme preterm (< 32 weeks); their main causes (preterm labor, prelabor rupture of membranes and therapeutic preterm birth) and treatments in all participant centers, which will enable us to evaluate more effective treatment strategies to adopt in these situations. The other study will be a nested case-control study with preterm births identified by researchers in the first months of data collection, having term births as controls (one control for each preterm birth), and analyzing risk factors for preterm birth.

### Sample size calculation

The sample size for the cross-sectional study was calculated using the Brazilian official preterm prevalence of around 6.5% [19]. Considering the acceptable absolute difference of 0.25% between the sample and the population prevalence, and a type I error of 5%, a sample size of 37,000 deliveries will be necessary [21]. To estimate the sample size related to the evaluation of risk factors in a nested case-control study, considering that the factors associated with preterm birth are highly heterogeneous, we had to choose one condition that, in most studies, was significantly associated with its occurrence. Therefore, using the estimate of smoking Brazilian pregnant women of approximately 20% [22], we choose to half this estimate to 10% to increase power. With an odds ratio of 1.74, a type I error (α) of 0.05 and a type II error (β) of 0.10, 1,014 pregnant women would be necessary in each group (with preterm and term births). An additional 4% increase in each group would guarantee that any lost of information would not compromise the final analysis. Therefore 1055 cases per group are planned.

Therefore, a total of 37,000 deliveries will be analyzed for a period of six months, considering the number of deliveries in the participant institutions. The total number of preterm births at the end of the study should be approximately 3,600.

### Variables

#### 1. Variables associated with the cross-sectional component

**Dependent variable**: preterm birth - every delivery that occurs before the 37^th ^week (less than 259 days) of pregnancy [WHO, 1976].

**Independent variables**: maternal age, skin color, marital status, level of education, family income, profession during pregnancy, number of pregnancies, parity, number of abortions, interval between pregnancies, use of licit or illicit drugs, partner's use of illicit drugs, physical activities during pregnancy, prenatal care, number of prenatal care visits, maternal history of chronic diseases, maternal clinical complications diagnosed during current pregnancy, spontaneous preterm labor, prelabor rupture of membranes, therapeutic preterm birth, polyhydramnio, congenital anomalies, stillbirth.

**Control variables**: place of origin, history of abortion, history of preterm labor, history of caesarian section, number of living children, usual maternal weight, maternal height, body mass index (BMI) at the end of pregnancy, weight gain during pregnancy, type of delivery, gestational age, newborn weight, adequacy of weight with gestational age, newborn vitality according to Apgar score, gender, gestational age, early neonatal morbidity, early neonatal mortality, late neonatal morbidity, late neonatal mortality.

#### 2. Variables associated with preterm birth causal conditions in the case-control component

They will be divided in three groups, according to the type of maternal complication.

**2.1. Dependent variable**: spontaneous preterm labor

**Independent variables**: rest, cerclage, number of uterine contractions, cervical dilation, cervical effacement, Bishop score, simple culture for group B *streptococcus*, selective culture for group B *streptococcus*, chorioamnionitis, maternal sepsis, oligohydramnios.

**2.2. Dependent variable**: prelabor rupture of membranes

**Independent variables**: diagnosis of prelabor rupture of membranes with visualization of amniotic fluid outflow, crystallization of amniotic fluid at microscope, vaginal pH, maternal hyperhidration, pulmonary hypoplasia, fetal anatomical deformities.

**2.3. Dependent variable**: therapeutic preterm labor

**Independent variables**: maternal condition that motivated pregnancy interruption, fetal condition that motivated pregnancy interruption, placental condition that motivated pregnancy interruption, elective interruption.

### Study population

**Figure 1:**
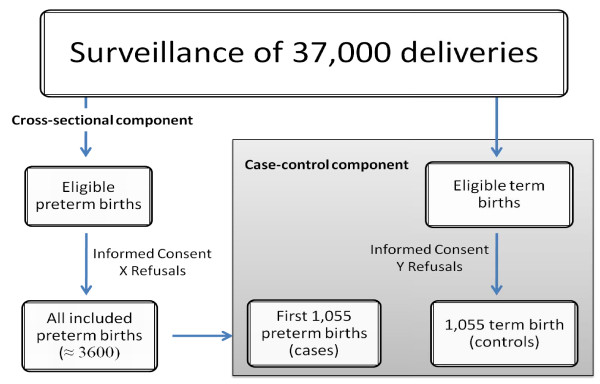
**Study design and population**.

The study population will comprehend all women who agree to participate and their preterm newborn infants, hospitalized during the data collection period, including twins and stillbirths. A group of women that give birth at term and are hospitalized in the institution during the first months of data collection, and who agree to participate in the study with their term newborn infants, will also be part of the study as a control group, including twins and stillbirths (Figure 1).

### Data collection procedures

Between the first and third days after delivery the woman who had preterm birth will be approached by the local researche assistant, who will explain the objectives of this study and inform her on the content of the Informed Consent. If she agrees to participate, she will receive a copy of the Informed Consent to read (if possible) and solve any questions and sign. She will then answer a questionnaire with the interest variables. This information will be supplemented with her medical chart and prenatal card. The newborn infant information will be obtained in his/her medical chart after he/she is discharged from hospital. For the control group, comprising women who give birth at term right after a woman who has preterm birth (first delivery that occurs at term after a preterm birth), considering one control for each case, the same procedures will be applied. Other information that may be of interest for the study but are not available in the medical charts will be obtained from the assistant medical team.

The causal conditions for preterm labor will be defined by each institution according to their own diagnosis criteria. The risk factors will be defined according to encoding of the variables above. The total number of deliveries at each institution during the period of study will also be informed.

Data will then be registered in a pre-encoded form especially developed for this study. A central database will be developed and data will be inserted into it through electronic forms by local researchers using the project's website.

### Data collection

Assistant researchers, called local coordinators, will receive live and online training to solve any doubts on the data collection process and the website before the beginning of collection. Also, they will be responsible for the analyses of the patients' medical charts in a daily basis, looking for preterm births and their respective controls. They will receive a Manual of Operation with instructions on how to complete questionnaires and forms. As soon as the woman who had preterm birth has been identified and signed the Informed Consent, she will be asked to complete a questionnaire, where situations such as preterm birth risks, pregnancy evolution, medical attention received, and type of delivery will be evaluated. Maternal medical charts will be analyzed before the patient is discharged from hospital, in order to clarify doubts on the aspects that may be of interest for the study. Newborn data will be collected using the babies' medical charts, which will be evaluated only after their discharge from hospital, transference or death, upon the mother's informed consent. In order to this procedure to be effective, every collaborating center should develop an administrative strategy to ensure adequate flow of medical charts. The procedure for the control group will be the same as that described for the case group. Children will not be followed. Only early neonatal information will be used.

Data will be inserted in an electronic form especially developed for this study, which will be provided to the participant centers by the coordinating center. After manual collection of data in the questionnaire, it will be inserted in electronic forms in the project website, hosted by Cemicamp's (Center for Studies in Reproductive Health of Campinas) institutional webpage and sent to the central database. Data missing in medical charts should be collected from other sources, such as hospital's database, prenatal cards, transference documents, etc. There will be a daily communication between the coordinating center and the participant centers during the data collection period.

Every center will have a restricted area in the site, where they will have access only to their own cases through a password. The global view of all cases in the Network will be in the form of monthly graphs and tables containing the number of cases included by each center and the distribution of referred diagnoses, provided by the coordinating center in the website's homepage.

In a monthly basis, the participating institutions will inform through the website the overall number of deliveries and preterm births that occurred in the previous month. This data will be checked by the main local researcher at the end of each month. In order to minimize doubts from researche assistants during the collection of data, a Manual of Operation will be developed with all information necessary to complete questionnaires and electronic forms, use the internet, access each center's database, standardize diagnostic definitions, among others.

A meeting will be held with all participant centers before the collection of data, in order to standardize the data collection process with the completion of questionnaires. A final meeting will be held with the local principal investigators at the end of the data collection process to discuss results, schedule final analyses, organize articles to be submitted for publication and define the responsibility and role of each person involved in this process.

### Data analysis plan

The data analysis will be carried out by subgroups according to the time of occurrence of the preterm birth and its determinant cause (spontaneous preterm labor, prelabor rupture of membranes, and therapeutic preterm birth). For the primary objectives the following will be estimated:

▪ Preterm birth rate by gestational age and frequency of causal condition;

▪ Classifications used by Institutions as diagnosis criteria to identify preterm birth causes, evaluating similarities and differences;

▪ Frequency of main maternal and fetal factors associated with preterm birth, calculating the odds ratio;

▪ Frequency of causal condition diagnostic procedures and type of adopted approach;

▪ Frequency of each type of medical attention during hospitalization and delivery;

▪ Frequency of most important neonatal results.

Initially, the overall prevalence of preterm birth will be calculated with a confidence interval (CI) of 95%. Preterm birth causes and gestational age interval will then be described according to their frequency. A bivariate analysis will be performed to evaluate possible individual risk factors for preterm birth, calculating the odds ratios with their respective CI of 95% [23,24]. Possible risk factors will be maternal sociodemographic variables, reproductive variables, maternal and fetal morbidity, as well as the characteristics of prenatal care and delivery. Finally, a multivariate analysis by logistic regression will be applied to jointly evaluate preterm birth risk factors, presenting the adjusted odds ratios and their respective 95%CI [25]. The PASW 17.0 program will be used for the statistical analysis of data.

### Quality control

Quality control procedures will be adopted, such as review of completed questionnaires, typing checking, consistency program, new collection of data from selected medical charts, and the use of a Manual of Operation. A data collection quality control will be first performed by the local researcher before and during the electronic input of forms, in order to identify possible inconsistencies of data. A second quality control will be performed using consistency programs. The principal researchers will temporarily visit the institutions to check the data.

The local researchers should maintain a record of problems occurred during the study and any doubt should be solved with the project's national coordinator.

### Study planning

In April of 2009, a meeting was held with the participant institutions during the Severe Maternal Morbidity study training, in the city of Campinas, São Paulo, when representatives from 27 Brazilian health institutions were present. The main aspects of the project were presented and the interested institutions were invited to participate in the national network on preterm studies. During May of 2009, a research proposal was developed and sent to the participant institutions. In June of 2009, the changes suggested by these institutions were implemented and confirmations to participate in the study were received.

### Ethical aspects

The collaborating centers will be definitely incorporated in the study only after the project has been approved by their respective Institutional Review Boards. The coordinating center will initially submit the proposal to its IRB, which will then submit it for approval to the National IRB. Women and newborn infants information will be obtained only after women have signed the written informed consent. All principles ruling research in human beings established by the Brazilian National Health Council Resolution 196/96 will be followed [26]. The confidentiality of women's data and medical attention will be ensured regardless of whether they participate in the study or not. The time necessary to complete each questionnaire will be approximately 20 minutes, and it will be performed after delivery while the patient is still in hospital. Complementary data of women and their newborn infants will be obtained from their respective medical charts.

## Discussion

### Technical and scientific contributions of the study

This study will provide a nationwide comprehensive evaluation of preterm births through the participation of health institutions with different regional characteristics. This will allow for the creation of a pioneer epidemiological database in the country, serving as a parameter to develop clinical trials and other studies on prevention, screening, diagnosis and treatment of pregnant women in risk of preterm labor, as well as the follow-up of cohorts of women and their preterm babies. There has been no such a huge multicenter collaboration between health institutions in Brazil to date, addressing this public health problem, neither with such a comprehensive obstetric and neonatal approach. In addition to this specific study on prevalence and risk factors, the organizational structure required by this project provides an ongoing investigation on the several conditions of public health interest, which are beyond the development period of this research. The development of an electronic data network with a specific database record and the commitment from renowned health institutions and national and global penetration in the scientific scenario is essential in a country with continental dimensions like Brazil. The participation of Pediatricians of national scope, with vast experience and scientific production in this area, as members of the Board will greatly contribute to improve the study, demonstrating the Obstetrics and Neonatology integration that is essential in studies on preterm birth.

Certainly, the availability of resources to implement and develop a National Network on Preterm Studies will bring umprecedent national and global scientific results, as well as the development of a pioneer technological basis to continuously obtain health data, providing necessary evidence for the real and effective improvement of the population's quality of life and health. This network, which is already committed to future initiatives on collaborative studies in perinatal and women health areas, should allow the implementation of a series of multicenter studies in perinatology that were never seen in this country, providing robust and nationwide results, which are essential to the great ethnic, cultural, and social diversity of the Brazilian population.

## Competing interests

The authors declare that they have no competing interests.

## Authors' contributions

All authors citaded above has made substantive intellectual contributions to the study, in all parts of them. All authors read and approved the final version of the manuscript.

## Pre-publication history

The pre-publication history for this paper can be accessed here:

http://www.biomedcentral.com/1471-2393/10/22/prepub
